# Post-marketing safety assessment of tetrandrine based on the WHO-VigiAccess database

**DOI:** 10.1097/MD.0000000000050823

**Published:** 2026-09-25

**Authors:** Yuanyuan Zhong, Xing Tan, Chunyue Fang

**Affiliations:** a Department of Pharmacy, The Third People’s Hospital of Yunnan Province, Kunming, Yunnan, China; b College of Pharmacy, Dali University, Dali, Yunnan, China.

**Keywords:** adverse drug reaction, adverse events, pharmacovigilance, tetrandrine, WHO-VigiAccess

## Abstract

Tetrandrine, a bisbenzylisoquinoline alkaloid extracted from *Stephania tetrandra* S. Moore, is clinically used in the treatment of silicosis and rheumatoid arthritis. Despite its demonstrated efficacy, systematic safety evaluations remain limited. This study represents the first effort to identify adverse event (AE) signals associated with tetrandrine meglumine using the World Health Organization VigiAccess database. Global AE reports for tetrandrine were extracted up to July 6, 2025. The collected data included demographic variables such as age group and gender, as well as regional distribution. Additionally, data on adverse drug reaction-related disease systems and symptoms were compiled from adverse drug reaction annual reports and reports submitted to the World Health Organization. Disproportionality analyses were conducted using the reporting odds ratio (ROR) and the Bayesian confidence propagation neural network. A total of 265 reports were included, documenting 448 AEs. The most commonly affected systems were gastrointestinal disorders (38.39%), skin and subcutaneous tissue disorders (16.96%), and nervous system disorders (16.07%). Frequently reported adverse reactions included nausea, dizziness, pruritus, vomiting, rash, abdominal pain, diarrhea, and abnormal hepatic function. Strong association signals were observed for oral anesthesia (ROR 241.19, 95% confidence interval [CI] 79.74–729.58), abnormal hepatic function (ROR 20.31, 95% CI 12.27–33.63), hypoaesthesia (ROR 10.47, 95% CI 4.66–23.54), pollakiuria (ROR 9.53, 95% CI 3.04–29.83), and abdominal pain (ROR 8.94, 95% CI 5.96–13.41). Additionally, potential adverse reactions not listed in the product labeling, including pruritus, rash, abnormal hepatic function, chest pain, palpitations, hypoaesthesia, edema, pyrexia, insomnia, pollakiuria, and dyspnea, were identified. This study comprehensively characterized the AE profile of tetrandrine and identified novel adverse effects. These findings underscore the importance of ongoing drug safety monitoring and patient management, and highlight the need for clinicians to use this medication more appropriately, tailoring treatment regimens based on the identified adverse effect profiles.

## 1. Introduction

Traditional Chinese medicines and their active ingredients have garnered increasing global attention due to their unique therapeutic effects and long historical usage, and they play a significant role in the treatment of various diseases. Tetrandrine, a bisbenzylisoquinoline alkaloid isolated from the roots of *Stephania tetrandra* S. Moore, is one of the major alkaloidal constituents.^[1]^ Tetrandrine is a calcium channel blocker,^[2]^ and exhibits a broad spectrum of pharmacological activities, including antifibrotic,^[3]^ anti-inflammatory,^[4,5]^ neuroprotective,^[6]^ antiviral,^[7]^ and anticancer effects.^[8]^ Studies have demonstrated that tetrandrine possesses considerable potential in the treatment of various in vitro solid tumor models, such as melanoma,^[9]^ lung cancer,^[10]^ and pancreatic cancer,^[11]^ among others.

Based on these properties, Chinese-developed preparations of tetrandrine, including tablets and injection, have been widely used in clinical practice for the treatment of silicosis, rheumatoid arthritis, and as adjuvant therapy for certain tumors.^[12]^ However, with the expansion of its clinical applications and prolonged usage, reports of potential safety risks, such as facial hyperpigmentation and abnormal hepatic function,^[13]^ have increasingly emerged, highlighting the urgent need for a comprehensive and systematic evaluation of its safety profile.

Ongoing monitoring and evaluation of drug safety is a critical component in ensuring public health safety. Premarket safety assessments primarily rely on clinical trials and animal experiments, which are conducted under controlled conditions that differ significantly from real-world clinical practice. As a result, these studies have notable limitations in detecting rare, delayed, or long-term adverse drug reactions (ADRs).^[14]^ Pharmacovigilance, particularly through spontaneous reporting systems (SRS), serves as a systematic and effective approach for monitoring and evaluating medication safety within public healthcare systems. Signal detection techniques based on SRS data have become essential tools for identifying potentially unknown safety risks associated with medications.^[15]^ The World Health Organization (WHO) has established VigiBase, the largest and most comprehensive international database of individual case safety reports. Its publicly accessible platform, VigiAccess, provides researchers with multiple avenues to identify and analyze ADRs. VigiAccess offers a valuable resource for retrieving and analyzing global ADR data. Data mining using VigiAccess enables more comprehensive and sensitive identification of adverse event (AE) signals potentially associated with specific drugs.

Currently, there is a relative lack of pharmacovigilance studies focusing on tetrandrine. This study aimed to systematically mine and analyze AE reports related to tetrandrine using the WHO-VigiAccess database. The findings provide an evidence-based reference for clinicians to ensure the safe use of tetrandrine methylin. It is expected that this study will fill the gap in systematic pharmacovigilance research on tetrandrine, offering a scientific basis for a deeper understanding of its safety profile, optimizing clinical decision-making regarding drug use, and ultimately safeguarding patient medication safety.

## 2. Materials and methods

### 2.1. Study design

This is a descriptive pharmacovigilance study utilizing individual case safety reports from VigiBase to perform disproportionality analysis and evaluate AE reports associated with tetrandrine. The research team employed rigorous methodologies to ensure the quality, validity, and reproducibility of the study findings.

### 2.2. Data sources

The analysis was conducted using VigiBase, the world’s largest AE database maintained by the WHO. VigiBase has been collecting safety reports since November 1967, and the dataset included information from multiple countries. This study was exempted from the approval of the Institutional Review Board, as it was an observational study based on fully anonymized patient data. It was carried out in accordance with pharmacovigilance guidelines (REporting of A Disproportionality analysis for drUg Safety signal detection using individual case safety reports in PharmacoVigilance).^[16]^ The WHO information system collected data on age group, sex, year of reporting, and major geographic regions worldwide.

### 2.3. Data extraction

Data were extracted as of July 6, 2025. The search was conducted using the generic name tetrandrine and the trade name JinAiKang, along with entry terms: hanjisong, isotetrandrine, and phaeanthine. All AE reports for this drug were retrieved. Since VigiAccess provides only aggregated summary data without access to individual case line listings, all retrieved reports matching the search criteria were included without further exclusion. The operational inclusion criteria were defined as all reports with tetrandrine recorded as the suspected/interacting drug, with no restrictions on age, sex, geographic region, or reporting year. No additional exclusion criteria were applied. The underlying VigiBase database is maintained by the WHO Uppsala Monitoring Centre, which applies standardized procedures for identifying and handling potential duplicate reports submitted from multiple sources. The aggregated data available through VigiAccess reflect these preprocessing and deduplication measures. Therefore, as users of the public VigiAccess platform, we did not perform additional duplicate detection or removal at the individual case level; all retrieved reports were included as provided by the system. Relevant demographic information was collected, including age group, sex, year of report, and geographic distribution by continent. We searched the records for tetrandrine and standardized the data using the system organ class (SOC) and preferred term (PT) from the Medical Dictionary for Regulatory Activities version 27.1 (MedDRA Maintenance and Support Services Organization, MSSO) to assess the range of toxicity.

### 2.4. Disproportionality analysis

Disproportionality analysis is commonly employed to explore potential associations between drugs and AEs within AE databases. In this study, the reporting odds ratio (ROR) method and the Bayesian confidence propagation neural network were utilized. ROR measures the degree of disproportionality in the reporting frequency of AEs associated with tetrandrine compared to other drugs.^[17]^ An AE signal related to tetrandrine is considered detected if the value is ≥ 3 and the lower limit of the 95% confidence interval (CI) is > 1. Bayesian confidence propagation neural network, based on Bayesian statistical principles, effectively addresses data sparsity and is particularly suitable for identifying signals of rare adverse reactions. An AE signal for tetrandrine was considered present if both a ≥ 3 and the lower confidence interval of information component (IC025) > 0 were satisfied. Meeting the criteria of both methods was interpreted as indicating a strong association between the drug and the AE. The dual-method analytical approach enhanced the robustness of signal detection. The formulas and evaluation criteria for both methods are presented in Table 1.

**Table 1:** ROR and BCPNN algorithms, equations, and criterias.

Algorithms	Equation	Criteria
ROR	$ROR=\frac{(a/c)}{(b/d)}=\frac{ad}{bc}$	a ≥ 3 95% CI (lower limit) > 1
	$95\;\%\;CI=e^{ln\left(ROR\right)\;\pm \;1\mathrm{.96}\sqrt{(\frac{1}{a}+\frac{1}{b}+\frac{1}{c}+\frac{1}{d})}}$	
BCPNN	$IC=log_{2}\frac{a(a+b+c+d)}{\left(a+b)(a+c\right)}$	a ≥ 3 IC025 > 0
	$\;\gamma \;=\gamma_{11}\frac{(a+b+c+d+\;\alpha \;)(a+b+c+d+\;\beta \;)}{(a+b+\alpha_{1})(a+c+\beta_{1})}$	
	$E\left(IC\right)=log_{2}\frac{(a+\gamma_{11})(a+b+c+d+\;\alpha \;)(a+b+c+d+\;\beta \;)}{(a+b+c+d+\;\gamma \;)(a+b+\alpha_{1})(a+c+\beta_{1})}$	
	$V\left(IC\right)=\frac{1}{{\left(ln2\right)}^{2}}$ $[\frac{\left(a+b+c+d\right)\text{-}a+\;\gamma \;\text{-}\gamma_{11}}{\left(a+\gamma_{11}\right)\left(1+a+b+c+d+\;\gamma \;\right)}+\frac{\left(a+b+c+d\right)\text{-}\left(a+b\right)+\;\alpha \;\text{-}\alpha_{1}}{\left(a+b+\alpha_{1}\right)\left(1+a+b+c+d+\;\alpha \;\right)}$ $+\frac{\left(a+b+c+d\right)\text{-}\left(a+c\right)+\;\beta \;\text{-}\beta_{1}}{(a+c+\beta_{1})(1+a+b+c+d+\;\beta \;)}]$	
	$IC025=E\left(IC\right)\text{-}2\sqrt{V(IC)}$	

a: the number of reports of specific drugs and specific adverse events; b: the number of reports of specific drugs and other adverse events; c: the number of reports for other drugs and specific adverse events; d: the number of reports of other drugs and other adverse events; γ, γ_11_ are the Dicichlet distribution parameter; α_1_, α, β_1_, β are the Beta distribution parameter; E(IC): the expectations of IC; V(IC): the variance of IC; IC025: the lower confidence interval of IC.

BCPNN = Bayesian confidence propagation neural network, CI = confidence interval, IC = information components, ROR = reporting odds ratio.

### 2.5. Data analysis

In this study, a retrospective quantitative research design was employed. The adverse reaction reporting proportion was calculated by dividing the total number of adverse reaction symptoms by the total number of adverse reaction reports. The reporting frequency of adverse reaction symptoms associated with the drug was computed and analyzed through comprehensive comparative methods. Descriptive variables were summarized using frequencies and percentages. Data processing and statistical analysis were performed using R software (version 4.4.3; R Foundation for Statistical Computing).

## 3. Results

### 3.1. Overall characteristics of adverse reactions reported for the target drugs

The first record of adverse reactions related to tetrandrine was documented in the WHO-VigiAccess database in 2014, with a total of 265 reports. Among these 265 adverse reaction reports associated with tetrandrine, the highest number was recorded in 2020, accounting for 27.17% (Fig. 1A). Gender information was available for all cases, and no obvious difference was observed between the number of ADRs reported for females (n = 151) and males (n = 114), as illustrated in Figure 1B. The male-to-female ratio was 1:1.3, indicating a relatively balanced gender distribution. Regarding age distribution, the highest reporting proportion was observed in the 45–64 years age group (Fig. 1C). Furthermore, all reported AEs occurred in Asia, representing 100% of the total. Additional details on the reporting years for the analyzed drugs are provided in Table 2.

**Table 2:** Characteristics of AEs for tetrandrine.

	Tetrandrine, n (%)
n	265
Female	151 (56.98)
Male	114 (43.02)
28 d–23 mo	1 (0.38)
12–17 yrs	2 (0.75)
18–44 yrs	51 (19.25)
45–64 yrs	123 (46.42)
65–74 yrs	65 (24.53)
≥ 75 yrs	23 (8.68)
Asia	265 (100.00)
2014	8 (3.02)
2015	18 (6.79)
2016	22 (8.30)
2017	30 (11.32)
2018	12 (4.53)
2019	47 (17.74)
2020	72 (27.17)
2021	31 (11.70)
2022	7 (2.64)
2023	11 (4.15)
2024	7 (2.64)

ADR = adverse drug reaction, AE = adverse event, n = number of ADR reports.

**Figure 1.**
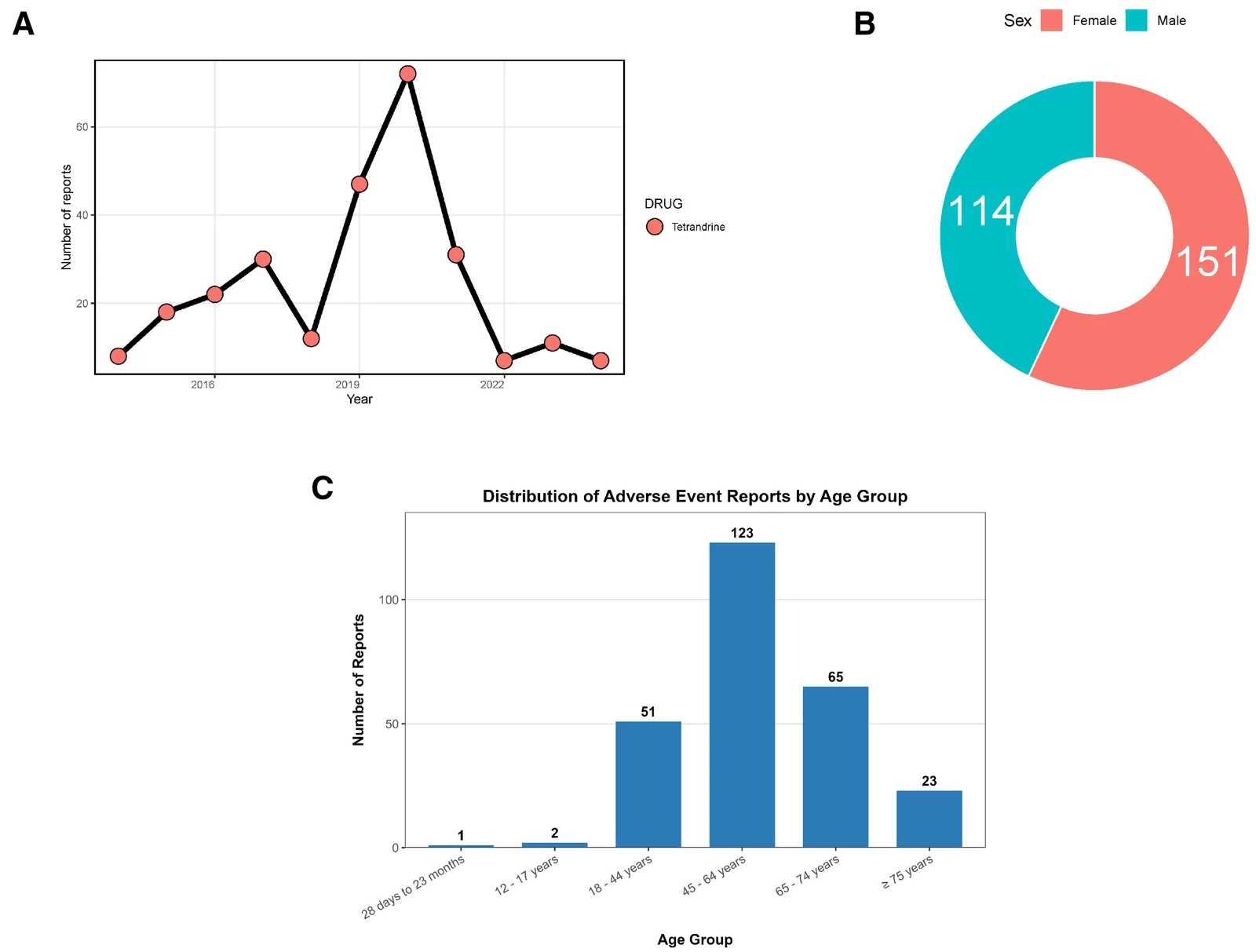
Overall characteristics of ADR reports for target drugs. (A) Annual trends in adverse drug event reports for tetrandrine; (B) gender distribution of reported events; (C) age distribution of reported events. ADR = adverse drug reaction.

### 3.2. Distribution of SOCs

AEs were categorized according to the SOC. Table S1, Supplemental Digital Content 1 presents the reporting frequencies of the 16 SOCs associated with tetrandrine. The drug showed a high reporting frequency for gastrointestinal disorders, skin and subcutaneous tissue disorders, and nervous system disorders. Among these, 4 SOCs had an ADR reporting proportion exceeding 10%. Figure 2 illustrates the distribution of these AEs at the SOC level.

**Figure 2.**
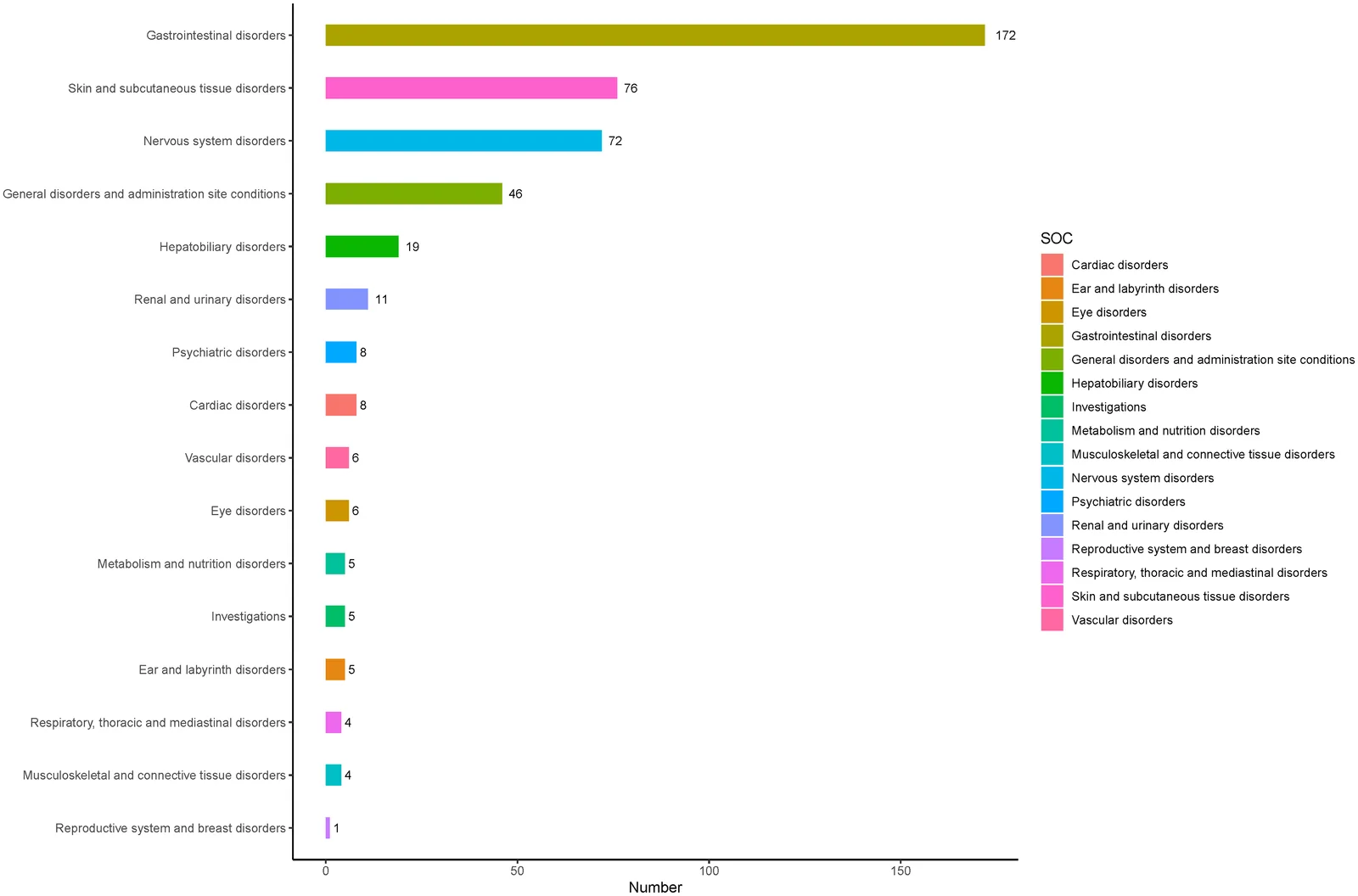
Percentage distribution of adverse reactions for tetrandrine in SOC. SOC = system organ class.

### 3.3. Analysis of emission signals at the PT Level

A total of 448 AEs were identified through screening for tetrandrine. The AEs associated with tetrandrine were categorized by frequency and evaluated for potential safety signals. At the PT level, the 30 most frequently reported AEs included nausea, dizziness, pruritus, vomiting, rash, abdominal pain, diarrhea, and hepatic function abnormal, among others. The AE profile of tetrandrine at the PT level is illustrated in Figure 3, showing the top 30 terms by frequency of occurrence. Higher signal intensity was observed for the following PTs: anesthesia oral (ROR 241.19, 95% CI 79.74–729.58), hepatic function abnormal (ROR 20.31, 95% CI 12.27–33.63), hypoaesthesia (ROR 10.47, 95% CI 4.66–23.54), pollakiuria (ROR 9.53, 95% CI 3.04–29.83), and abdominal pain (ROR 8.94, 95% CI 5.96–13.4), as presented in Table 3. Additionally, potential adverse reactions not listed in the product labeling were identified, including pruritus, rash, hepatic function abnormal, chest pain, palpitations, hypoaesthesia, edema, pyrexia, insomnia, pollakiuria, and dyspnea.

**Table 3:** All AEs of tetrandrine with positive signals at the PT level.

PT	Case numbers	ROR (95% Cl)	IC (IC025)
Anaesthesia oral	4	241.19 (79.74–729.58)	7.56 (6.11)
Hepatic function abnormal	16	20.31 (12.27–33.63)	4.26 (3.54)
Hypoaesthesia	6	10.47 (4.66–23.54)	3.36 (2.25)
Pollakiuria	3	9.53 (3.04–29.83)	3.23 (1.77)
Abdominal pain	25	8.94 (5.96–13.4)	3.07 (2.49)
Chest pain	14	8.49 (4.97–14.49)	3.03 (2.27)
Abdominal distension	7	7.09 (3.35–15)	2.8 (1.76)
Dizziness	40	6.03 (4.35–8.35)	2.47 (2)
Vertigo	3	5.39 (1.72–16.82)	2.41 (0.96)
Vomiting	29	5.31 (3.64–7.75)	2.33 (1.78)
Nausea	54	5 (3.76–6.65)	2.17 (1.76)
Abdominal discomfort	14	4.3 (2.52–7.33)	2.06 (1.31)
Flushing	3	4.15 (1.33–12.94)	2.04 (0.59)
Hyperhidrosis	3	3.98 (1.28–12.42)	1.98 (0.53)
Palpitations	6	3.91 (1.74–8.76)	1.95 (0.85)
Pruritus	30	3.84 (2.65–5.56)	1.86 (1.33)
Asthenia	14	3.38 (1.99–5.77)	1.72 (0.96)
Headache	13	2.78 (1.6–4.83)	1.44 (0.66)
Somnolence	9	2.25 (1.16–4.37)	1.15 (0.23)
Diarrhoea	21	2.04 (1.31–3.16)	0.99 (0.36)
Rash	26	2 (1.34–2.97)	0.95 (0.38)

IC025: the lower confidence interval of IC.

AE = adverse event, CI = confidence interval, IC = information components, PT = preferred term, ROR = reporting odds ratio.

**Figure 3.**
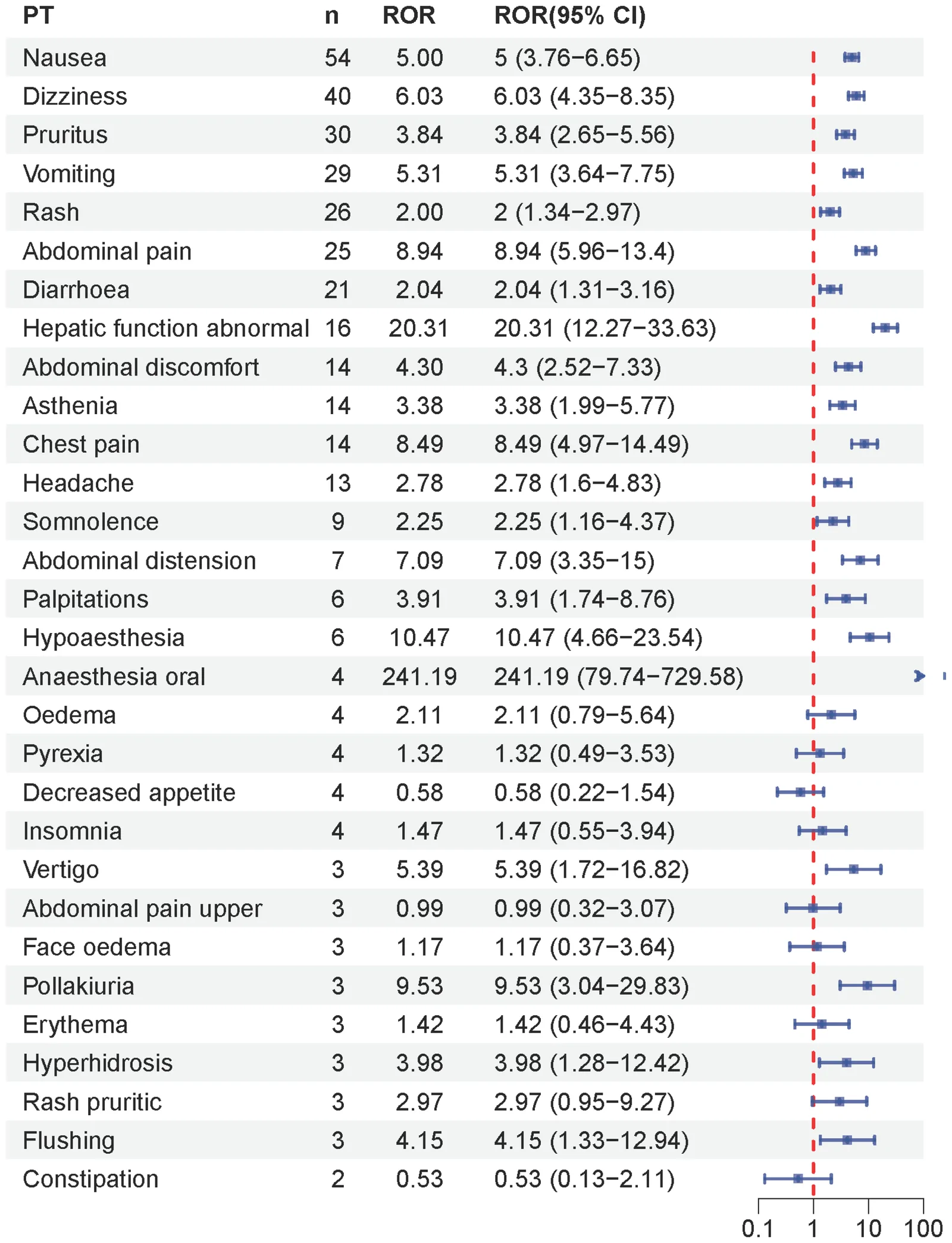
Top 30 adverse reactions by frequency for tetrandrine at the PT level. PT = preferred term.

## 4. Discussion

It is widely recognized that ADRs and drug safety are of paramount importance, as ADRs can reduce patient compliance and even lead to treatment interruption. A better understanding of AEs is essential to prevent or at least minimize them and to facilitate their recognition in clinical practice. However, to date, studies investigating AEs induced by tetrandrine using real-world data remain limited.

Tetrandrine is a plant extract that has been approved for the clinical treatment of silicosis in China,^[18,19]^ and has been used for decades with documented clinical efficacy. Tetrandrine can modulate a variety of signaling molecules, such as cell cycle kinases, rat sarcoma pathway kinases, and oncogene proteins.^[20]^ In addition, tetrandrine can reverse drug resistance by binding to the active site of P-glycoprotein,^[21]^ which is another advantage of tetrandrine.

With the rapid development of its clinical applications and the advancement of long-term follow-up studies, its safety and tolerability are continuously being evaluated and improved. Like any therapeutic drug, tetrandrine may cause ADRs ranging from mild to severe, necessitating careful monitoring in both clinical studies and real-world practice.

In this study, we comprehensively assessed relevant AEs reported between 2014 and 2024 by analyzing VigiAccess data. Descriptive analyses showed that common adverse reactions included nausea, abdominal discomfort, asthenia, and somnolence, which are well documented in the product labeling. However, our analysis also identified potential adverse reactions not listed in the product labeling, such as pruritus, rash, abnormal hepatic function, chest pain, palpitations, hypesthesia, edema, pyrexia, insomnia, pollakiuria, and dyspnea. This highlights the importance of continuous post-marketing surveillance in identifying rare or late-onset adverse reactions.

The age distribution revealed that the highest percentage of ADRs was found in the 45 to 64 years age group. Patients in this group are typically middle-aged or older, often with comorbidities, decreased immune function, and genetic variability in pharmacokinetics and pharmacodynamics. Therefore, the reporting proportion of ADRs appeared to be higher in this age group.^[22]^ Additionally, geographic distribution data showed that ADRs were reported exclusively from Asia, likely due to the widespread use of the drug in China. The drug is not currently approved by the US Food and Drug Administration for sale as a prescription or over-the-counter drug in the United States. However, there are reports of clinical trials of tetrandrine for the treatment of relapsed/refractory leukemia in the United States, suggesting a potential for future use outside of Asia.

Tetrandrine exhibited the highest reported rate of adverse reactions (38.39%) in the gastrointestinal disorders category, primarily including nausea, vomiting, abdominal pain, and diarrhea. Severe gastrointestinal reactions can significantly reduce therapeutic efficacy and patient compliance. Treatment of diarrhea primarily relies on empirical symptomatic therapy, and patient education is essential. Dietary advice that may reduce the likelihood of diarrhea (e.g., ensuring low fiber intake) should be provided at the initiation of treatment. Patients should also be encouraged to increase fluid intake to prevent dehydration. In terms of therapeutic options, probiotics can be used to regulate intestinal flora and alleviate diarrhea.^[23]^

Adverse reactions associated with tetrandrine and skin and subcutaneous tissue disorders in this study accounted for 16.96%, mainly characterized by pruritus (6.70%) and rash (5.80%). Dermatotoxicity, such as rash and pruritus, is a common treatment-related side effect, usually manifesting as rashes on the face, neck, and upper chest, and is closely related to the pharmacologic effects of the drug. The exact mechanism of dermatotoxicity induced by tetrandrine remains unclear. Some studies have shown that tetrandrine inhibits Wnt/β-catenin activity by blocking β-catenin nuclear translocation, decreasing Wnt3a levels, and modulating key downstream targets such as CyclinD1 and c-Myc.^[24]^ Based on the drug’s antitumor and immune-modulatory effects, it may cause skin rashes.^[25]^ This may be due to the fact that tetrandrine inhibits cell growth and survival, activates the mitochondrial apoptotic pathway, induces apoptosis through inhibition of the phosphatidylinositol 3-kinase/protein kinase B/mammalian target of rapamycin pathway,^[26]^ inhibits adhesion to the host microenvironment, inhibits angiogenesis,^[27]^ and enhances immune cell activity. Additionally, tetrandrine’ s immunomodulatory properties, particularly its capacity to modulate T-cell activity and cytokine release, may contribute to the inflammatory cutaneous responses observed in some patients.^[28,29]^ However, these mechanistic hypotheses remain speculative and require direct experimental confirmation. Some or all of these effects may also affect keratinocytes in patients treated with tetrandrine and contribute to the development of rash. Preventive measures, such as regular use of emollients (alcohol-free) from the beginning of treatment, use of mild soaps, and avoidance of temperature extremes and direct sunlight, may help prevent or reduce the severity of rash.^[30]^ Mild to moderate rashes can be treated with topical corticosteroids and/or oral antihistamines.

In this study, tetrandrine was found to cause abnormal hepatic function (3.57%), which is an adverse reaction not listed in the product labeling. Acceleration of tetrandrine has been found to cause hepatic injury in some animal models. Li et al hypothesized that the interaction of tetrandrine with p38α mitogen-activated protein kinase resulted in liver injury, while other studies suggested that cytochrome P450 (CYP) enzymes were involved in tetrandrine-induced hepatotoxicity.^[31]^ Qi et al suggested that CYP2E1 was the main cause of mitochondrial dysfunction in rat hepatocytes after exposure to tetrandrine, which is related to reactive oxygen species generated during CYP2E1 metabolism.^[32]^ It has been shown that ursolic acid attenuates tetrandrine-induced hepatotoxicity by competitively binding to the substrate-binding site of glutathione S-transferase and is a novel antidote to attenuate tetrandrine-induced hepatic injury.^[33]^ Therefore, the hepatic function of patients should be carefully evaluated, and early identification and prophylactic measures should be implemented.

The pathophysiologic mechanisms behind tetrandrine-induced hypoaesthesia, which appears to be strongly associated with oral anesthesia and hypoaesthesia in nervous system disorders, are not fully understood. It may involve direct neurotoxicity, modulation of central monoaminergic neurotransmitter systems, or disruption of the blood-brain barrier. Some findings show that tetrandrine elevated 5-hydroxytryptamine levels in the hippocampus of norepinephrine and mice and significantly increased brain-derived neurotrophic factor protein levels and messenger RNA expression in rat hippocampus.^[34]^ Altered levels of neurotransmitters may affect the neural pathways responsible for sensory transmission, whereas sympathetic activation may inhibit oral blood flow and sensory signaling, further contributing to diminished oral sensation.^[35]^ In addition, metabolic or nutritional deficiencies, including vitamin B12, folic acid, or other micronutrients, can lead to peripheral neuropathy, which in turn can cause sensory abnormalities. Even in the short term, clinicians should be aware of this potential AE and consider alternative treatments when necessary. In addition to the serotonergic and neurotrophic mechanisms, tetrandrine’ s well-established calcium channel-blocking activity may directly influence neuronal excitability and sensory transmission. Voltage-gated calcium channels play a critical role in neurotransmitter release and membrane depolarization in sensory neurons; their modulation by tetrandrine could theoretically lead to altered peripheral sensory perception, manifesting as hypoaesthesia or oral anesthesia.^[2]^ Furthermore, the ability of tetrandrine to cross the blood-brain barrier raises the possibility of direct central nervous system involvement. The strikingly strong signal for oral anesthesia warrants particular attention; we hypothesize that it may reflect a local anesthetic-like effect of tetrandrine on peripheral sensory nerve endings, possibly through interference with voltage-gated sodium or calcium channels that are essential for action potential generation and propagation. This hypothesis is biologically plausible given that tetrandrine shares structural features with other bisbenzylisoquinoline alkaloids known to interact with ion channels.

This study is limited by the inherent bias of SRS. First, there may have been underreporting or selective reporting, potentially underestimating the true incidence of ADRs. Second, the lack of clinical variables, such as exact dose, treatment duration, and concomitant medication status, may have hindered risk stratification and confounded ADR attribution. Specifically, VigiAccess does not provide individual-level data on dose titration, cumulative exposure, or co-medication, which precludes any dose-response analysis or assessment of potential drug-drug interactions in the current study. These important questions should be addressed in future investigations using the complete VigiBase database (with appropriate authorization) or through dedicated prospective cohort studies. In addition, pharmacovigilance databases lack consistent data on whether ADRs occur with a single drug or in combination, limiting the assessment of the independent safety of each drug. Finally, these limitations restrict our ability to assess causality and validate proposed mechanisms. Further clinical studies are needed to provide higher-level evidence.

## 5. Conclusion

This study provides a comprehensive overview of the adverse reaction profile associated with tetrandrine, which predominantly affects the gastrointestinal, cutaneous, neurological, and hepatic systems. It highlights the importance of individualized monitoring during treatment to enhance drug safety. The implementation of optimized adverse reaction management strategies can further improve treatment safety. Further clinical studies are warranted to better understand the long-term safety and efficacy of tetrandrine.

## Author contributions

**Conceptualization:** Yuanyuan Zhong, Xing Tan.

**Data curation:** Chunyue Fang.

**Funding acquisition:** Yuanyuan Zhong.

**Methodology:** Yuanyuan Zhong, Xing Tan.

**Resources:** Chunyue Fang.

**Software:** Yuanyuan Zhong.

**Visualization:** Xing Tan, Chunyue Fang.

**Writing – original draft:** Yuanyuan Zhong.

**Writing – review & editing:** Yuanyuan Zhong.



## References

[1] Chan WC Zhao Q Wong KH. Tetrandrine regulates NAADP-mediated calcium signaling through a LIMP-2-dependent and sphingosine-mediated mechanism. Nat Commun. 2025;16:6308. 10.1038/s41467-025-61565-9 PMC12238253 40628771

[2] Liu QY Karpinski E Pang PK. Tetrandrine inhibits both T and L calcium channel currents in ventricular cells. J Cardiovasc Pharmacol. 1992;20:513–9. 10.1097/00005344-199210000-00001 1280704

[3] Ma P Cai YJ Dong H Dong RL. Research progress on anti-fibrotic effects and mechanisms of tetrandrine. Chin J Integr Med. 2025;31:837–45. 10.1007/s11655-025-4130-4 40407855

[4] Lu S Wang P Hu J Zhang Z. Tetrandrine improves severe acute pancreatitis by inhibiting NCOA4 glycosylation-mediated binding to FTH1 and inducing autophagy-dependent ferroptosis. FASEB J. 2025;39:e70592. 10.1096/fj.202500404R 40326888

[5] Wu Y Wang XQ Wu JY. A tri-compound formula comprising Ginsenoside Rg1, tetrandrine and icariin alleviates atopic dermatitis symptoms in a mouse model. Phytomedicine. 2025;141:156737. 10.1016/j.phymed.2025.156737 40222169

[6] Wang W Li Y Li Y Zhao YM Ye JB Qian T. Tetrandrine mediates autophagy via sirtuin 3/adenosine 5-monophosphate-activated protein kinase/mammalian target of rapamycin signal pathway to attenuate early brain injury after subarachnoid hemorrhage. Neuroreport. 2025;36:514–23. 10.1097/WNR.0000000000002171 PMC12133048 40377960

[7] Liu J Wang F Wang X. Antiviral effects and tissue exposure of tetrandrine against SARS-CoV-2 infection and COVID-19. MedComm (2020). 2023;4:e206. 10.1002/mco2.206 PMC9851407 36699286

[8] Xin G Hou Y Liu Y Li H. Tetrandrine and adriamycin reverse multidrug resistance by regulating NLRP3/Caspase-1/GSDMD signaling in human breast cancer cells. Eur J Pharmacol. 2025;998:177635. 10.1016/j.ejphar.2025.177635 40252903

[9] He LN Liu YJ Jiang JB. Tetrandrine augments melanoma cell immunogenicity via dual inhibition of autophagic flux and proteasomal activity enhancing MHC-I presentation. Acta Pharmacol Sin. 2025;46:2056–72. 10.1038/s41401-025-01507-9 PMC12205077 40016522

[10] Fang Z Lin P Gao R Yang W Zhou A Yu W. Preparation, characterization, and anti-lung cancer activity of tetrandrine-loaded stealth liposomes. Int J Nanomedicine. 2024;19:787–803. 10.2147/IJN.S431599 PMC10825470 38293606

[11] Wang Y Xu T Wang H Xia G Huang X. Inhibition of autophagy induced by tetrandrine promotes the accumulation of reactive oxygen species and sensitizes efficacy of tetrandrine in pancreatic cancer. Cancer Cell Int. 2024;24:241. 10.1186/s12935-024-03410-5 PMC11238362 38987818

[12] Guo X Qi J Li H Xing Z. Clinical efficacy of acetylcysteine combined with tetrandrine tablets on patients with silicosis and its effect on exercise tolerance and pulmonary function. Exp Ther Med. 2020;20:1285–90. 10.3892/etm.2020.8858 PMC7388567 32765668

[13] Zhou S Shi J Chen Z Bian L Huang L Mao L. Tetrandrine slows disease progression on high-resolution computed tomography and lung function decline in artificial stone-associated silicosis: a retrospective cohort study. BMC Pulm Med. 2025;25:343. 10.1186/s12890-025-03821-8 PMC12276650 40684152

[14] Hu H Zhao Y Mao J. A real-world pharmacovigilance study of adverse drug reactions associated with lecanemab and aducanumab based on WHO-VigiAccess and FAERS databases. Front Pharmacol. 2025;16:1561020. 10.3389/fphar.2025.1561020 PMC12023478 40290431

[15] JeongESuYLiLChenY. Discovering clinical drug-drug interactions with known pharmacokinetics mechanisms using spontaneous reporting systems and electronic health records. J Biomed Inform. 2024;153:104639.10.1016/j.jbi.2024.10463938583580

[16] Fusaroli M Salvo F Begaud B. The REporting of A Disproportionality Analysis for DrUg Safety Signal Detection Using Individual Case Safety Reports in PharmacoVigilance (READUS-PV): explanation and elaboration. Drug Saf. 2024;47:585–99. 10.1007/s40264-024-01423-7 PMC11116264 38713347

[17] Li L Bai J Wen X Zeng X. Adverse reactions of four multi-targeted tyrosine kinase inhibitors: a descriptive analysis of the WHO-VigiAccess database. Front Pharmacol. 2025;16:1585862. 10.3389/fphar.2025.1585862 PMC12052882 40331199

[18] Song MY Wang JX Sun YL. Tetrandrine alleviates silicosis by inhibiting canonical and non-canonical NLRP3 inflammasome activation in lung macrophages. Acta Pharmacol Sin. 2022;43:1274–84. 10.1038/s41401-021-00693-6 PMC9061833 34417574

[19] Banks DE Cheng YH Weber SL Ma JK. Strategies for the treatment of pneumoconiosis. Occup Med. 1993;8:205–32. 8456348

[20] Bhagya N Chandrashekar KR. Tetrandrine and cancer - an overview on the molecular approach. Biomed Pharmacother. 2018;97:624–32. 10.1016/j.biopha.2017.10.116 29101806

[21] Lima EN Lamichhane S Bahadur KCP Ferreira ES Koul S Koul HK. Tetrandrine for targeting therapy resistance in cancer. Curr Top Med Chem. 2024;24:1035–49. 10.2174/0115680266282360240222062032 PMC11259026 38445699

[22] Gurwitz JH Field TS Harrold LR. Incidence and preventability of adverse drug events among older persons in the ambulatory setting. JAMA. 2003;289:1107–16. 10.1001/jama.289.9.1107 12622580

[23] Benson AB 3rd Ajani JA Catalano RB. Recommended guidelines for the treatment of cancer treatment-induced diarrhea. J Clin Oncol. 2004;22:2918–26. 10.1200/JCO.2004.04.132 15254061

[24] Tian H Wang P Shao H. Natural bioactive compounds targeting the Wnt/β-catenin pathway for the treatment of hepatocellular carcinoma. J Hepatocell Carcinoma. 2025;12:1613–22. 10.2147/JHC.S523261 PMC12296707 40718077

[25] Tinsley SM Kurtin SE Ridgeway JA. Practical management of lenalidomide-related rash. Clin Lymphoma Myeloma Leuk. 2015;15(Suppl):S64–9. 10.1016/j.clml.2015.02.008 26297281

[26] Ling J Li X Wang M. Novel sulfonyl-substituted tetrandrine derivatives for colon cancer treatment by inducing mitochondrial apoptosis and inhibiting PI3K/AKT/mTOR pathway. Bioorg Chem. 2024;143:107069. 10.1016/j.bioorg.2023.107069 38160477

[27] Zhao H Kong L Shen J. Tetrandrine inhibits the occurrence and development of frozen shoulder by inhibiting inflammation, angiogenesis, and fibrosis. Biomed Pharmacother. 2021;140:111700. 10.1016/j.biopha.2021.111700 34044279

[28] Niu L Chang J Liu S. Tetrandrine induces cell cycle arrest in cutaneous melanoma cells by inhibiting IL-6/CDC42 signaling. Arch Dermatol Res. 2025;317:442. 10.1007/s00403-025-03930-8 39976634

[29] Wang K Deng J Yang J. Tetrandrine promotes the survival of the random skin flap via the PI3K/AKT signaling pathway. Phytother Res. 2024;38:527–38. 10.1002/ptr.8058 37909161

[30] De Wit M Boers-Doets CB Saettini A. Prevention and management of adverse events related to regorafenib. Support Care Cancer. 2014;22:837–46. 10.1007/s00520-013-2085-z PMC3913844 24337717

[31] Li F Dong YZ Zhang D Zhang XM Lin ZJ Zhang B. Molecular mechanisms involved in drug-induced liver injury caused by urate-lowering Chinese herbs: a network pharmacology study and biology experiments. PLoS One. 2019;14:e0216948. 10.1371/journal.pone.0216948 PMC6541264 31141540

[32] Qi XM Miao LL Cai Y Gong LK Ren J. ROS generated by CYP450, especially CYP2E1, mediate mitochondrial dysfunction induced by tetrandrine in rat hepatocytes. Acta Pharmacol Sin. 2013;34:1229–36. 10.1038/aps.2013.62 PMC4003161 23892269

[33] Chu S Lu Y Liu W. Ursolic acid alleviates tetrandrine-induced hepatotoxicity by competitively binding to the substrate-binding site of glutathione S-transferases. Phytomedicine. 2022;104:154325. 10.1016/j.phymed.2022.154325 35820303

[34] Gao S Cui YL Yu CQ Wang QS Zhang Y. Tetrandrine exerts antidepressant-like effects in animal models: role of brain-derived neurotrophic factor. Behav Brain Res. 2013;238:79–85. 10.1016/j.bbr.2012.10.015 23085478

[35] Templeton D Thulin A. Secretory, motor and vascular effects in the sublingual gland of the rat caused by autonomic nerve stimulation. Q J Exp Physiol Cogn Med Sci. 1978;63:59–66. 10.1113/expphysiol.1978.sp002415 244363

