# Supplementary material for: Post-marketing safety assessment of tetrandrine based on the WHO-VigiAccess database
Source: Medicine (Baltimore). 2026 Sep 25;105(39):e50823. doi: 10.1097/MD.0000000000050823 (PMC13619188; doi:10.1097/MD.0000000000050823)
Supplement: Supplementary file 1 [file medi-105-e50823-s001.docx]

Supplementary Table S1. Number and reporting proportion of adverse reactions across 16 SOCs for tetrandrine.

| SOC | Tetrandrine(N=448) |
| --- | --- |
| Gastrointestinal disorders | 172(38.39%) |
| Skin and subcutaneous tissue disorders | 76(16.96%) |
| Nervous system disorders | 72(16.07%) |
| General disorders and administration site conditions | 46(10.27%) |
| Hepatobiliary disorders | 19(4.24%) |
| Renal and urinary disorders | 11(2.46%) |
| Cardiac disorders | 8(1.79%) |
| Psychiatric disorders | 8(1.79%) |
| Vascular disorders | 6(1.34%) |
| Eye disorders | 6(1.34%) |
| Investigations | 5(1.12%) |
| Metabolism and nutrition disorders | 5(1.12%) |
| Ear and labyrinth disorders | 5(1.12%) |
| Respiratory, thoracic and mediastinal disorders | 4(0.89%) |
| Musculoskeletal and connective tissue disorders | 4(0.89%) |
| Reproductive system and breast disorders | 1(0.22%) |

SOC: System organ class.
